# Extended Kalman Filter-Based Methods for Pose Estimation Using Visual, Inertial and Magnetic Sensors: Comparative Analysis and Performance Evaluation

**DOI:** 10.3390/s130201919

**Published:** 2013-02-04

**Authors:** Gabriele Ligorio, Angelo Maria Sabatini

**Affiliations:** The Institute of BioRobotics, Scuola Superiore Sant'Anna, Piazza Martiri della Libertà 33, 56124 Pisa, Italy; E-Mail: angelo.sabatini@sssup.it

**Keywords:** sensor fusion, extended Kalman filtering, inertial/magnetic sensing, monocular vision, ego-motion

## Abstract

In this paper measurements from a monocular vision system are fused with inertial/magnetic measurements from an Inertial Measurement Unit (IMU) rigidly connected to the camera. Two Extended Kalman filters (EKFs) were developed to estimate the pose of the IMU/camera sensor moving relative to a rigid scene (ego-motion), based on a set of fiducials. The two filters were identical as for the state equation and the measurement equations of the inertial/magnetic sensors. The DLT-based EKF exploited visual estimates of the ego-motion using a variant of the Direct Linear Transformation (DLT) method; the error-driven EKF exploited pseudo-measurements based on the projection errors from measured two-dimensional point features to the corresponding three-dimensional fiducials. The two filters were off-line analyzed in different experimental conditions and compared to a purely IMU-based EKF used for estimating the orientation of the IMU/camera sensor. The DLT-based EKF was more accurate than the error-driven EKF, less robust against loss of visual features, and equivalent in terms of computational complexity. Orientation root mean square errors (RMSEs) of 1° (1.5°), and position RMSEs of 3.5 mm (10 mm) were achieved in our experiments by the DLT-based EKF (error-driven EKF); by contrast, orientation RMSEs of 1.6° were achieved by the purely IMU-based EKF.

## 1. Introduction

Sensor fusion methods combine data from disparate sources of information in a way that should ideally give better performance than that achieved when each source of information is used alone. The design of systems based on sensor fusion methods requires the availability of complementary sensors in order that the disadvantages of each sensor are overcome by the advantages of the others. An interesting application niche for sensor fusion—the one dealt with in this paper—is motion tracking. None of the several existing sensor technologies, taken alone, can meet the desired performance specifications, especially when motion is to be tracked without restrictions in space and time [1]. Vision and inertial/magnetic sensors are considered in this regard a particularly useful combination for developing a sense of position (localization) and motion, which is critically important in several technical fields, including augmented reality [2,3], robotics [4–7] and human machine interfaces [8].

Vision-based tracking systems can accurately track the relative motion between the camera and objects within its field of view (FOV) by measuring the frame-by-frame displacements of selected features, such as points or lines [9]. The camera pose relative to the scene can be estimated in all six degrees of freedom (DOFs) by using a stereo-camera system or by incorporating some a priori knowledge of the scene when a monocular system is used. The information provided by finding and associating image points of interest through a monocular video stream (monocular visual tracking) can be used to estimate the camera orientation relative to an absolute reference frame. The concurrent estimation of environment structure and motion allows to recover the perception of depth, otherwise lost from a single perspective view, using multiple images taken from different viewpoints [9]. The main shortcoming of vision-based tracking systems is the slow acquisition rate, which is due to both the physics of the image acquisition process and the computational workload of the computer-vision algorithms, especially those used to detect the visual features in each image frame. The consequence is that vision-based tracking systems lack robustness against fast motion dynamics, which may easily lead to loss of visual features. Another difficulty with vision-based tracking systems is that the line of sight between the camera and objects within its FOV must be preserved as much as possible, in other words vision-based tracking systems are severely prone to problems of occlusions.

Inertial-based tracking systems integrate Inertial Measurement Units (IMUs) that incorporate accelerometers and gyroscopes for measuring translational accelerations and angular velocities of the objects they are affixed to with high sampling rates; this feature makes them ideally suited to capture fast motion dynamics. Being internally referenced and immune to shadowing and occlusions, inertial sensors can track body motion, in principle, without restrictions in space. Unfortunately, measurements of linear accelerations and angular velocities are affected by time-varying bias and wideband measurement noise of inertial sensors. Accurate estimates of body orientation in the three-dimensional (3D) space can be produced using quite complex filtering algorithms, sometimes with the addition of magnetic sensors that sense the Earth's magnetic field to help producing drift-free heading estimates [10]; conversely, the 3D body position can be accurately estimated in tracking systems operating in a single IMU configuration only within temporally limited intervals of time, unless specific motion constraints are known and exploited to mitigate the double-time integration errors of gravity-compensated measured accelerations. The latter approach has been successfully implemented in strap-down inertial navigation systems (INS) for applications of pedestrian navigation [11,12].

Fusing visual and inertial/magnetic measurements can therefore yield, in principle, a tracking system for pose estimation in all six DOFs that retains, at the same time, the long-term stability and the accuracy of a vision-based tracking system with the short-term robustness and promptness of response typical of an INS [13]. Two main approaches have been tried to exploit the complementary properties of visual and inertial sensors, namely the loosely coupled approach and the tightly coupled approach [13]. In the loosely coupled approach [14–16], the vision-based tracking system and the INS exchange information each other, while the sensor data processing takes place in separate modules. The information delivered by the IMU can be used to speed up the tracking task of the features by predicting their locations within the next frame; in turn, data from the visual sensor allows updating the calibration parameters of inertial sensors. Conversely, in the tightly coupled approach all measurements, either visual or inertial, are combined and processed using a statistical filtering framework. In particular, Kalman filter-based methods are the preferred tool to perform sensor fusion [2,17,18].

In this paper the problem of estimating the ego-motion of a hand-held IMU-camera system is addressed. The presented development stems from our ongoing research on tracking position and orientation of human body segments for applications in telerehabilitation. While orientation tracking can be successfully performed using EKF-based sensor fusion methods based on inertial/magnetic measurements [10,19,20], position tracking requires some form of aiding [21].

A tightly coupled approach was adopted to the design of a system in which pose estimates were derived from observations of fiducials. Two EKF-based sensor fusion methods were developed that built somewhat upon the approaches investigated in [2,18], respectively. They were called DLT-based EKF (DLT: Direct Linear Transformation) and error-driven EKF. Their names were intended to denote the different use made of visual information available from fiducials: the visually estimated pose produced by the DLT method was directly delivered to the DLT-based EKF, while in the error-driven EKF the visual measurements were the difference between the measured and predicted location of the fiducials in the image plane. In each filter 2D frame-to-frame correspondences were established by a process of model-based visual feature tracking: a feature was searched within a size-variable window around its predicted location, based on 3D known coordinates of fiducials and the *a priori* state estimate delivered by the EKF. Moreover, the visual measurement equations were stacked to the measurement equations for the IMU sensors (accelerometer and magnetic sensor), and paired to the state transition equation, where the state vector included quaternion of rotation, position and velocity of the body frame relative to the navigation frame.

The main contributions of this paper are: (a) the comparative analysis and performance evaluation of the two different forms of visual aiding—the study was extended to the case when visual and inertial/magnetic measurements were used alone; (b) the investigation of the role played by magnetic sensors and related measurements of the Earth's magnetic field for heading stabilization, never attempted before in research on visuo-inertial integration (to the best of our knowledge). This paper is organized as follows: Section 2 reports the description of our experimental setup and a detailed mathematical analysis of the filtering methods. Main results achieved so far are presented in Section 3 and then discussed in Section 4. Finally, we offer concluding remarks and perspectives for our future work in Section 5.

## 2. Methods

**Figure 1.**
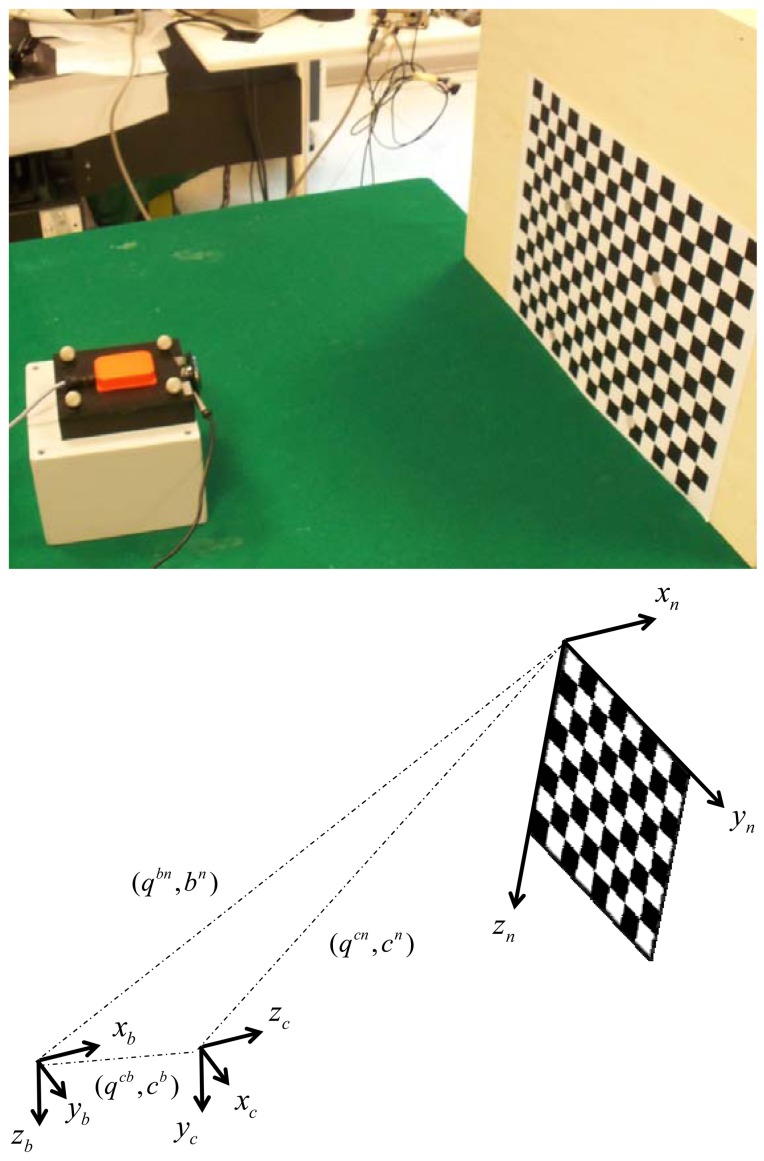
The camera is rigidly attached to the same support where the IMU sensor case is also attached. The axes of the three frames {**n**}, {**c**} and {**b**} are also drawn.

We introduce the reference frames that are used in the experimental setup shown in Figure 1:

- Navigation frame {**n**}—this is the frame in which the coordinates of the corner points of a chessboard are known and the Earth's gravity and magnetic fields are assumed known, or measurable. The goal of the sensor fusion methods is to estimate the pose of the IMU case, namely the body pose, in {**n**}.
- Body frame {**b**}—this frame is attached to the IMU case, and the inertial and magnetic measurements delivered by the IMU are resolved in {**b**}.
- Camera frame {**c**}—this frame is attached to the camera, with its origin located in the camera optical center and the Z-axis pointing along the optical axis; although the camera is rigidly connected with the IMU, {**c**} is different from {**b**}.
- Image frame {**i**}—the 2D coordinate frame of the camera images; it is located in the image plane, which is perpendicular to the optical axis.

The following notation is used to express the relation between two frames, for instance {**c**} and {**b**}: ${\text{R}}^{cb}\text{ and }{\bar{\text{q}}}^{cb}={\left[{\left({\text{q}}^{cb}\right)}^{T}q_{4}^{cb}\right]}^{T}$ denote, respectively, the rotation matrix and the quaternion from {**b**} to {**c**} (***q̅****^cb^* is the vector part and $q_{4}^{cb}$ is the scalar part of ***q̅****^cb^*, [22]); ***b****^c^* represents the position of {**b**} relative to {**c**}.

Figure 1 shows the sensor unit assembly and the chessboard. The sensor unit assembly contains one web-cam and one IMU; they are housed in a plastic box and are rigidly connected to each other. The visual sensor is a Microsoft web-cam with resolution 640 × 480 that acquires black-and-white visual images at approximately 30 fps; the images are transferred to the host computer via a USB port. The time elapsed between the time instant when the acquisition process starts and the time instant when a new image frame is available is returned together with the visual data.

The IMU is an MTx orientation tracker (Xsens Technologies B.V., Enschede, The Netherlands) equipped with one tri-axial accelerometer, one tri-axial gyroscope and one tri-axial magnetic sensor, with mutually orthogonal sensitive axes; the raw sensory data are delivered to the host computer at 100 Hz via another USB port. Both the camera and the IMU are electrically synchronized to an optical motion analysis system Vicon 460 equipped with six infrared (IR) cameras running at 100 Hz. The 3D coordinates of eight IR-reflective markers are acquired. Four markers (diameter: 15 mm; are located at the corners of the plastic box housing the sensor unit assembly, and four markers of the same diameter are located on the chessboard plane, where they are used for capturing the 3D coordinates of four black-and-white extreme corners of the chessboard. Since the size of the chessboard printed on an A3 sheet of paper is known, the 3D coordinates resolved in {**n**} of each black-and-white corner of the chessboard are easily determined.

The ancillary laboratory frame where the 3D positions of the markers are given is used to compute the transformation from {**b**} to {**n**}, yielding the reference data ${\text{R}}_{\text{ref}}^{nb}$ or ${\bar{\text{q}}}_{\text{ref}}^{nb}$, and ${\text{b}}_{\text{ref}}^{n}$ that are needed for assessing the performance of the proposed sensor fusion methods. As for the IMU-camera relative pose calibration problem, namely the estimation of the rigid body transformation from {**c**} to {**b**}, **R***^cb^* or ***q̅****^cb^* are determined using the method proposed in [23]; the translation vector ***b****^c^* is determined using a ruler, since accurate knowledge of this quantity is not critically important, especially when tracking slow motions [2].

### 2.1. Purely IMU-Based Method of Orientation Estimation

The purely IMU-based method for determining the IMU orientation relative to {**n**} revolves around the EKF developed in [10]. The major difference is that neither gyro bias nor magnetic distortions are included in the state vector for self-compensation purposes: the state vector ***x***_R_*_k_* = ***x***_R_(*t_k_*) is simply composed of the quaternion ***q̅****^nb^* sampled at the time instants *t_k_*. The suffix R stands for rotation, to indicate the components of the state vector that describe the rotational behavior of the IMU-camera sensor unit assembly relative to {**n**}, see below. The angular velocity ***ω*** = [*p q r*]*^T^* measured by the gyroscopes is used to update the state vector according to the state-transition model:

(1) $${\text{x}}_{\text{R}\;k}={\text{F}}_{\text{R}\;k-1}{\text{x}}_{\text{R}\;k-1}+{\text{w}}_{\text{R}\;k-1}$$

The rotational state transition matrix **F**_R_ *_k_*_–1_ is related to ***ω*** as follows:

(2) $${\text{F}}_{\text{R}\;k-1}=\text{exp}[\Omega (\omega_{k-1})\Delta_{k}]$$

where Δ*_k_* = *t_k_* − *t_k_*_–1_ is the sampling interval and **Ω**(***ω***) is the operator:

(3) $$\Omega (\omega )=\left[\begin{array}{cc} \left[\omega \times \right] & \omega \\ -\omega^{T} & 0 \end{array}\right]$$

and [***ω*** ×] is the skew-symmetric operator, [22]:

(4) $$\left[\omega \times \right]=\left[\begin{array}{ccc} 0 & -r & q\\ r & 0 & -p\\ -q & p & 0 \end{array}\right]$$

The process noise vector ***w***_R_*_k_*_–1_ is related to the noise in the angular velocity measurements as follows:

(5) wRk−1=Δk2⋅[[qk−1nb×]+I3⋅q4,k−1nb−qk−1nb]vk−1g=Δk2⋅Ξ(q¯k−1nb)vk−1g

where ^*g*^***v****_k_*_–1_ is the gyroscope measurement noise, which is assumed white Gaussian with zero mean and covariance matrix $\Sigma_{g}={\text{I}}_{3}\cdot \sigma_{g}^{2}$ (**I***_n_* is the *n* × *n* identity matrix). The process noise covariance matrix can be shown to have the following expression [10]:

(6) $${\text{Q}}_{\text{R}\;k-1}={\left(\frac{\Delta_{k}}{2}\right)}^{2}\cdot \Xi \left({\bar{\text{q}}}_{k-1}^{nb}\right)\Sigma_{g}\Xi {\left({\bar{\text{q}}}_{k-1}^{nb}\right)}^{T}$$

When tracked motions are relatively slow, as it is assumed in this paper, the sensed acceleration is simply taken as the projection of the gravity ***g****^n^* along the sensitivity axes of the tri-axial accelerometer.

Since no heading information is available when the gravity vector is sensed, the measurement of the Earth's magnetic field ***h****^n^* by the magnetic sensor may help producing drift-free heading estimates. The measurement equations are written as:

(7) hkb=Rkbnhn+vkh={(q¯knb)−1⊗h¯n⊗q¯knb}V+vkhakb=Rkbngn+vka={(q¯knb)−1⊗g¯n⊗q¯knb}V+vka

where *^h^****v****_k_* and *^a^****v****_k_* are the measurement noises superimposed to the output of the accelerometer and the magnetic sensor, respectively; they are assumed white Gaussian with zero mean and covariance matrices $\Sigma_{h}={\text{I}}_{3}\cdot \sigma_{h}^{2}$ and $\Sigma_{a}={\text{I}}_{3}\cdot \sigma_{a}^{2}$, respectively. The operator ⊗ in Equation (7) is the quaternion product, ***q̅***^−1^ denotes the quaternion inverse, and ***h̅****^n^* and ***g̅****^n^* are quaternions with zero scalar part and vector part ***h̅****^n^* and ***g̅****^n^*, respectively. The operator {***q̅***}*_V_* denotes the vector part of the quaternion ***q̅***.

The EKF linearization requires the computation of the Jacobian matrices of the measurement Equation (7):

(8) $$\begin{array}{c} {\text{H}}_{k}^{\text{mag}}=\Psi \left({\bar{\text{q}}}_{k}^{nb},{\text{h}}^{n}\right)=\frac{\partial \left({\left({\bar{\text{q}}}_{k}^{nb}\right)}^{-1}⊗{\bar{\text{h}}}^{n}⊗{\bar{\text{q}}}_{k}^{nb}\right)}{\partial {\bar{\text{q}}}_{k}^{nb}}\\ {\text{H}}_{k}^{\text{acc}}=\Psi \left({\bar{\text{q}}}_{k}^{nb},{\text{g}}^{n}\right)=\frac{\partial \left({\left({\bar{\text{q}}}_{k}^{nb}\right)}^{-1}⊗{\bar{\text{g}}}^{n}⊗{\bar{\text{q}}}_{k}^{nb}\right)}{\partial {\bar{\text{q}}}_{k}^{nb}} \end{array}$$

The operator **Ψ**(***q̅***, ***p***) can be written for the quaternion ***q̅*** = [*q*_1_ *q*_2_ *q*_3_ *q*_4_]*^T^* and a quaternion ***p̅*** with vector part ***q*** and zero scalar part as follows [22]:

(9) $$\Psi \left(\bar{\text{q}},\text{p}\right)={\left\{\bar{\text{q}}\right\}}_{\text{R}}{\left\{\bar{\text{p}}\right\}}_{\text{R}}^{T}+{\left\{\bar{\text{q}}\right\}}_{\text{L}}^{T}{\left\{\bar{p}\right\}}_{\text{L}}\left[\begin{array}{cc} -{\text{I}}_{3} & 0\\ 0 & 1 \end{array}\right]$$

where:

(10) $${\left\{\bar{\text{q}}\right\}}_{\text{L}}=\left[\begin{array}{cccc} q_{4} & q_{3} & -q_{2} & q_{1}\\ -q_{3} & q_{4} & q_{1} & q_{2}\\ q_{2} & -q_{1} & q_{4} & q_{3}\\ -q_{1} & -q_{2} & -q_{3} & q_{4} \end{array}\right]{\left\{\bar{\text{q}}\right\}}_{\text{R}}=\left[\begin{array}{cccc} q_{4} & -q_{3} & q_{2} & q_{1}\\ q_{3} & q_{4} & -q_{1} & q_{2}\\ -q_{2} & q_{1} & q_{4} & q_{3}\\ -q_{1} & -q_{2} & -q_{3} & q_{4} \end{array}\right]$$

The measurement noise covariance matrix is given by:

(11) $$\text{R}=\left[\begin{array}{cc} \Sigma_{\text{h}} & 0_{3}\\ 0_{3} & \Sigma_{a} \end{array}\right]$$

where **0***_n_* is the *n* × *n* null matrix. In order to guard against the effect of spurious magnetic measurements, which can be produced especially in indoor environments where magnetic fields are far from being homogeneous, the vector selection technique proposed in [24] is implemented: the strength of the sensed magnetic field*h*normand the dip angle*θ*_dip_, namely the angle that is formed between the sensed magnetic field and the sensed gravity acceleration, are compared to their nominal values using suitably chosen threshold values,*λ*_dip_ and *λ_h_*, respectively. Whenever either difference exceeds the corresponding threshold value, the magnetic measurement is considered invalid and therefore it is discarded from the filtering process by setting the matrix ${\text{H}}_{k}^{\text{mag}}$ to zero. A similar vector selection technique is implemented by comparing the norm of the measured acceleration vector *a*_norm_ with the value of gravity (1*g* = 9.81 m/s^2^) [19]: the acceleration measurement vector is assimilated by the EKF only when the absolute difference between *a*_norm_ and *g* is less than a threshold value *λ_g_*, otherwise ${\text{H}}_{k}^{\text{acc}}$ is set to zero.

### 2.2. Purely Vision-Based Method of Pose Estimation

**Figure 2.**
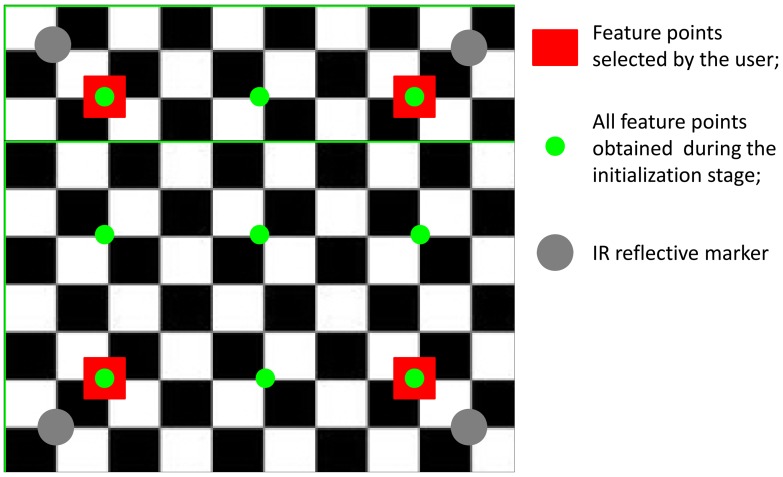
The nine feature points constructed during the initialization stage are shown. Red squares: the four feature points manually selected by the user; green circles: the nine feature points constructed during the initialization stage.

We assume that the visual features are the projections into the image plane of *N_f_* chessboard corners (*N_f_* = 9) which represents our fiducial markers (Figure 2). Initially the user is asked to click on the four extreme corners of the chessboard in the first image frame, starting from the upper-left corner and proceeding counterclockwise; five additional 3D/2D correspondences are established by projecting the 3D chessboard model available in {**n**} back to the image plane in {**i**} based on the homography estimated using the four features selected by the user. The nine image point features we choose to identify in the first frame are then tracked using the pyramidal implementation of the Kanade-Lucas tracker (KLT) [25–27]. Henceforth, the squared area whose vertices are the four extreme corners of the chessboard is called the chessboard area.

The image point features are fed to a least-squares estimation algorithm to calculate the transformation from {**n**} to {**c**} [28]. This algorithm is a variant of the DLT method [29], suited for tracking plane surfaces like the chessboard. The covariance matrix of the estimated pose is computed at each iteration step by analyzing the projection errors of the feature image points as suggested in [9].

### 2.3. EKF-Based Sensor Fusion Methods of Pose Estimation

The EKF-based sensor fusion method of body pose estimation requires that the rotational state vector ***x***_R_ = ***q̅****^nb^* is extended using the components of the translational state vector ${\text{x}}_{\text{T}}={\left[\begin{array}{cccccc} {\text{b}}_{x}^{n} & {\dot{b}}_{x}^{n} & {\text{b}}_{y}^{n} & {\dot{b}}_{y}^{n} & {\text{b}}_{z}^{n} & {\dot{b}}_{z}^{n} \end{array}\right]}^{T}$ which includes the position ${\text{b}}^{n}={\left[\begin{array}{ccc} {\text{b}}_{x}^{n} & {\text{b}}_{y}^{n} & {\text{b}}_{z}^{n} \end{array}\right]}^{T}$ and velocity ${\dot{\text{b}}}^{n}={\left[\begin{array}{ccc} {\dot{b}}_{x}^{n} & {\dot{b}}_{y}^{n} & {\dot{b}}_{z}^{n} \end{array}\right]}^{T}$ of the IMU case in {**n**}. The state-transition model equation is given by:

(12) $${\text{x}}_{\text{T}\;k}={\text{F}}_{\text{T}\;k-1}{\text{x}}_{\text{T}k-1}+{\text{w}}_{\text{T}\;k-1}$$

In our approach accelerometers are used for stabilizing the IMU-camera attitude with respect to gravity (roll and pitch angles), as prescribed by the measurement Equation (7), under the assumption that the magnitude of the gravity vector is large enough to dominate the body acceleration, which is modeled as noise:

(13) $${\ddot{\text{b}}}^{\text{n}}=\text{w}\;$$

where ***w*** is white Gaussian noise, with zero mean and covariance matrix *^a^***Σ** = **I**_3_ · *^a^σ*^2^, where the variance *^a^σ*^2^ is also called the strength of the driving noise [30].

The state transition matrix can be written as:

(14) $${\text{F}}_{\text{T}\;k}=\left[\begin{array}{ccc} \left[\begin{array}{cc} 1 & \Delta_{k}\\ 0 & 1 \end{array}\right] & 0_{2} & 0_{2}\\ 0_{2} & \left[\begin{array}{cc} 1 & \Delta_{k}\\ 0 & 1 \end{array}\right] & 0_{2}\\ 0_{2} & 0_{2} & \left[\begin{array}{cc} 1 & \Delta_{k}\\ 0 & 1 \end{array}\right] \end{array}\right]$$

where Δ*_k_* is the time interval elapsed between successive measurements, regardless of which sensors produce them.

The noise covariance matrix of the process noise ***w***_T_*_k_*_–1_ can be written as:

(15) QTk=[[Δk4/4Δk3/2Δk3/2Δk2]020202[Δk4/4Δk3/2Δk3/2Δk2]020202[Δk4/4Δk3/2Δk3/2Δk2]]σ2a

The simplifying assumption that the translational and rotational components of the body motion are uncoupled is then made in writing the state transition model of the overall state vector ${\text{x}}_{k}={\left[\begin{array}{cc} {\text{x}}_{\text{R}\;k}^{T} & {\text{x}}_{\text{T}\;k}^{T} \end{array}\right]}^{T}$ as follows:

(16) $${\text{x}}_{k}=\left[\begin{array}{cc} {\text{F}}_{\text{R}\;\;k-1} & 0_{4\times 6}\\ 0_{6\times 4} & {\text{F}}_{\text{T}\;k-1} \end{array}\right]{\text{x}}_{k-1}$$

The covariance matrix of the process noise ${\text{w}}_{k-1}={\left[{\text{w}}_{\text{R}\;k-1}^{T}{\text{w}}_{\text{T}\;k-1}^{T}\right]}^{T}$ is:

(17) $${\text{Q}}_{k-1}=\left[\begin{array}{cc} {\text{Q}}_{\text{R}\;k-1} & 0_{4\times 6}\\ 0_{6\times 4} & {\text{Q}}_{\text{T}\;k-1} \end{array}\right]$$

**Figure 3.**
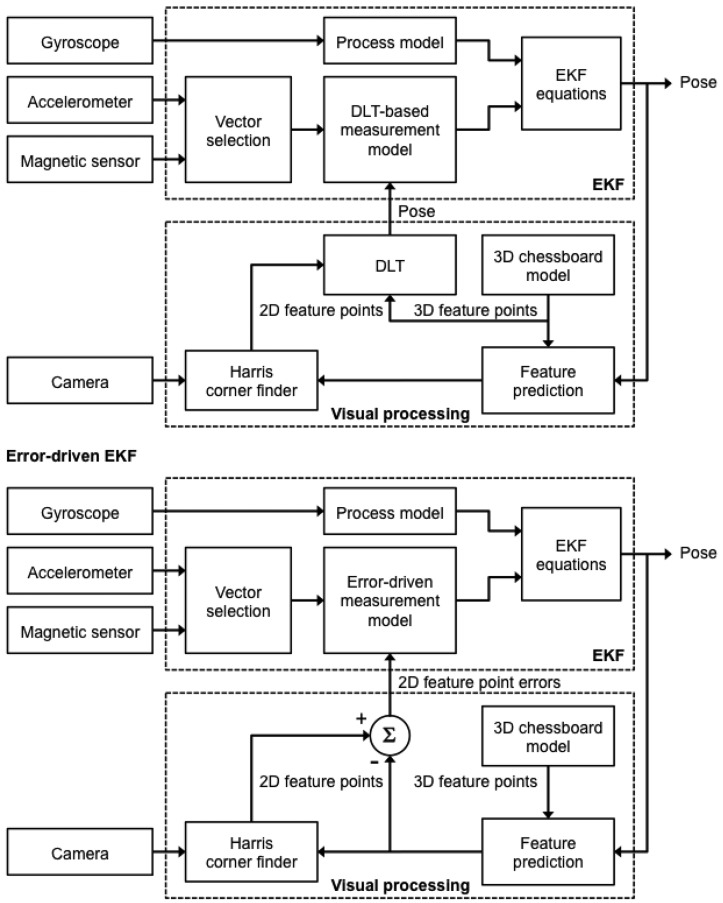
Block diagrams of the two Kalman-filter-based methods of sensor fusion.

Two different sensor fusion strategies are considered to account for how to add the visual measurements ${\text{y}}_{k}^{\text{vis}}$ to Equation (7), which leads to different dependencies between the output variables and the components of the system's state vector. Henceforth the two measurement models are called the *DLT-based model* and the *error-driven model*, hence the name DLT-based EKF and error-driven EKF for the corresponding sensor fusion methods, see Figure 3.

A common element to both methods is the approach to visual feature tracking. While the purely vision-based method of pose estimation relies on the popular frame-to-frame KLT, visual feature tracking in either the DLT-based EKF or the error-driven EKF exploits the predicted *a priori* point features ${\hat{\text{p}}}_{j,k}^{i}\;j=1,\ldots ,N_{f}$ that are obtained from the projection of the 3D chessboard model in {**i**}:

(18) $${\hat{\text{p}}}_{j,k}^{i}=\text{K}\;{\text{R}}^{c\text{b}}\left({\hat{\text{R}}}_{k}^{bn}\left({\text{Z}}_{j}^{n}-{\hat{\text{b}}}_{k}^{n}\right)-{\text{b}}^{c}\right),j=1,\ldots ,N_{f}$$

where ${\hat{\text{R}}}_{k}^{bn}$ and ${\hat{\text{b}}}_{k}^{n}$ are derived from the *a priori* estimate of the state vector, and **K** is the camera calibration matrix [9]:

(19) $$\text{K}=\left[\begin{array}{ccc} f_{x} & \beta & -x_{c}\\ 0 & f_{y} & -y_{c}\\ 0 & 0 & 1 \end{array}\right]$$

*f_x_* and *f_y_* are the two components of the focal length (theoretically, they should be equal), *β* takes accounts for any pixel misalignment within the optical sensor, while *x*_c_ and *y*_c_ are the coordinates of the principal point (image centre) relative to the origin of the frame {**i**}. Equation (18) is based on the “pinhole model”, according to which an ideal planar lens is assumed and optical distortion is neglected. Actually, the image point features are compensated for the distortion introduced by the lens system using the so-called Brown-Conrady model [31]. All camera intrinsic parameters, involved both in the camera calibration matrix and in the distortion model, were estimated during the camera calibration stage [32].

Features points ${\hat{\text{p}}}_{j,k}^{i}$ are then used as initial conditions for the Harris corner finder. The Harris corner finder works by searching for the nearest black-and-white corner within a window that is centered around its predicted location [33]. The search window size, which is constrained between 5 and 20 pixels, is adaptively computed based on the predicted *a priori* error covariance.

For either method, the overall linearized measurement model can be written in the following form:

(20) $${\text{H}}_{k}=\left[\begin{array}{cc} {\text{H}}_{k}^{\text{mag}} & 0_{3\times 6}\\ {\text{H}}_{k}^{\text{acc}} & 0_{3\times 6}\\ {\text{H}}_{\text{R}k}^{\text{vis}} & {\text{H}}_{\text{T}k}^{\text{vis}} \end{array}\right]$$

The measurement noise covariance matrix is written as follows:

(21) $${\text{R}}_{k}=\left[\begin{array}{ccc} {\text{R}}^{\text{mag}} & 0_{3} & 0_{3\times N_{\text{vis}}}\\ 0_{3} & {\text{R}}^{\text{acc}} & 0_{3\times N_{\text{vis}}}\\ 0_{N_{\text{vis}}\times 3} & 0_{N_{\text{vis}}\times 3} & {\text{R}}_{k}^{\text{vis}} \end{array}\right]$$

The size of the matrices **H***_k_* and **R***_k_* depends on which EKF-based sensor fusion method we consider. Implicit in the formulation of Equation (20) is that inertial/magnetic sensing contributes only to the estimate of orientation, while visual sensing conveys information about all the six DOFs.

**Figure 4.**
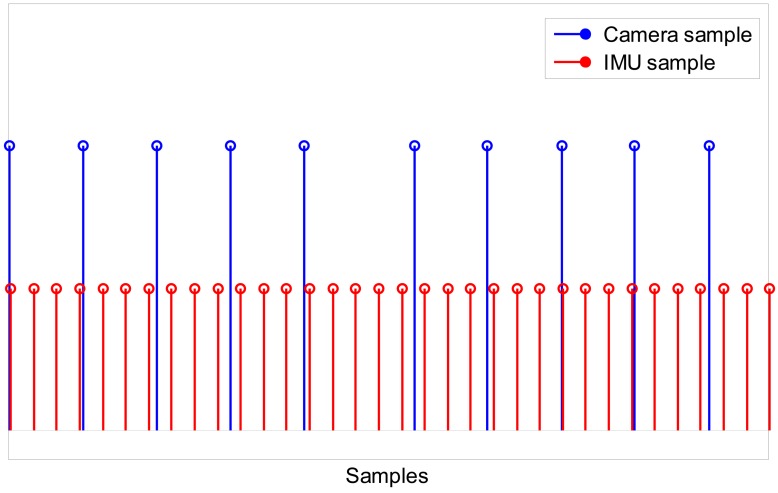
Timestamps of camera (blue) and IMU (red) samples. The number of IMU samples between successive image frames is slightly variable due to the irregular sampling rate of the camera.

A multi-rate filtering strategy is needed to deal with the different sampling rates of IMU and camera measurements: the IMU measurement process runs at a rate of 100 Hz, while the camera measurement process is slower, running at a rate of approximately 30 fps (Figure 4). Both EKFs can be defined as multi-rate, which alludes to the transition between different measurement equations that must be performed within the filter depending on which measurements are available. Since the time instant when the inertial/magnetic and visual measurements are made is known to the system, the time lag between successive measurements Δ*_k_* is also known, which allows propagating the state vector in the prediction stage and selecting which rows of the Jacobian matrix in Equation (20) would be actually set to zero in the update stage at any iteration step of the filter. In other words, in the time intervals between successive image frames from the camera only IMU measurements are to be processed, which implies that the measurement equations of both EKFs are identical to the measurement equations of the purely IMU-based method of orientation determination described in Section 2.1. Then, when a new image frame becomes available, the measurement equations are suitably changed in order to assimilate the visual information, leading to the measurement equations presented in Sections 2.3.1 and 2.3.2 for the two EKFs (see below).

#### 2.3.1. DLT-Based Measurement Model

The DLT method reviewed in Section 2.2 provides, for each incoming image frame, the estimate of the chessboard pose in {**c**}, in terms of ${\text{R}}_{k}^{cn}$ or ${\bar{\text{q}}}_{k}^{cn}$ and ${\text{n}}_{k}^{c}$. This is based on using the correspondences between the image point features and their corresponding corner points on the chessboard. The DLT output can be expressed directly in terms of the body pose in {**n**} using the following transformations:

(22) $$\begin{array}{l} {\bar{\text{q}}}_{k}^{nb}={\left({\bar{\text{q}}}_{k}^{cn}\right)}^{-1}⊗{\bar{\text{q}}}^{c\text{b}}\\ {\text{b}}_{k}^{n}={\left({\text{R}}_{k}^{cn}\right)}^{T}({\text{b}}^{c}-{\text{n}}_{k}^{c}) \end{array}$$

We recall that ***q̅***^cb^ and ***b***^c^ are known from solving the IMU-camera relative pose calibration problem, as already described above.

The visual observation matrix can be simply written as:

(23) $${\text{H}}^{\text{vis}}=\left[{\text{H}}_{R}^{\text{vis}}{\text{H}}_{T}^{\text{vis}}\right]=\left[\begin{array}{cc} {\text{I}}_{4} & 0_{4\times 6}\\ 0_{6\times 4} & \left[\begin{array}{cccccc} 1 & 0 & 0 & 0 & 0 & 0\\ 0 & 0 & 1 & 0 & 0 & 0\\ 0 & 0 & 0 & 0 & 1 & 0 \end{array}\right] \end{array}\right]$$

The measurement noise covariance matrix is:

(24) $${\text{R}}_{k}^{\text{vis}}=\left[\begin{array}{cc} \text{cov}\left({\bar{\text{q}}}_{k}^{n\text{b}}\right) & 0_{4\times 3}\\ 0_{3\times 4} & \text{cov}\left({\text{b}}_{k}^{n}\right) \end{array}\right]$$

where:

(25) $$\begin{array}{l} \text{cov}\left({\bar{\text{q}}}_{k}^{nb}\right)=\frac{1}{4}\Xi \left({\bar{\text{q}}}_{k}^{nb}\right)\text{cov}\left(\psi ,\vartheta ,\phi \right)\Xi^{\text{T}}\left({\bar{\text{q}}}_{k}^{nb}\right)\\ \text{cov}\left({\text{b}}_{k}^{n}\right)={\left({\text{R}}_{k}^{cn}\right)}^{T}\text{cov}({\text{n}}_{k}^{c}){\text{R}}_{k}^{cn} \end{array}$$

In principle, the covariance matrix cov(*ψ, ϑ, ϕ*) of the Euler angles *ψ, ϑ, ϕ* and the covariance matrix $\text{cov}({\text{n}}_{k}^{c})$ of the translation vector ${\text{n}}_{k}^{c}$ are provided by the DLT method using the methods described in [9]. However, a stable behavior of the DLT-based EKF is simply obtained by taking $\text{cov}\left(\psi ,\vartheta ,\phi \right)={\text{I}}_{3}\cdot \sigma_{\theta }^{2}\left(\sigma_{\theta }=0.05{}^{\circ}\right)$ and $\text{cov}\left({\text{b}}_{k}^{n}\right)={\text{I}}_{3}\cdot \sigma_{b}^{2}\left(\sigma_{b}=1\;\text{mm}\right)$. These values are in close agreement with the experimental uncertainty estimated during extensive experimental testing of the DLT method in our experimental setup (not reported in this paper).

#### 2.3.2. Error-Driven Measurement Model

The feature projection errors at time *t_k_* are the difference between the measured image point features with coordinates ${\text{z}}_{j,k}^{i},j=1,\ldots ,N_{f}$ and the a priori predicted features points ${\hat{\text{p}}}_{j,k}^{i}$ (see Section 2.3).

The measurement equation can be written as:

(26) $${\text{y}}_{j,k}^{\text{vis}}=\text{K}\;{\text{R}}^{cb}\left({\hat{\text{R}}}_{k}^{bn}\left({\text{Z}}_{j}^{n}-{\hat{\text{b}}}_{k}^{n}\right)-{\text{b}}^{c}\right)-{\text{z}}_{j,k}^{i}$$

Since the dependence of the measurements ${\text{y}}_{j,k}^{\text{vis}}$ from the quaternion ${\bar{\text{q}}}_{k}^{nb}$ is nonlinear, the Jacobian matrix of the transformation from Equation (26) must be computed as part of the EKF linearization:

(27) $$\frac{\partial {\text{y}}_{j,k}^{\text{vis}}}{\partial {\bar{\text{q}}}_{k}^{n\text{b}}}=\text{K}\;{\text{R}}^{c\text{b}}\frac{\partial \left({\left({\bar{\text{q}}}_{k}^{nb}\right)}^{-1}⊗\bar{{\text{Z}}_{j}^{n}-{\text{b}}_{k}^{n}}⊗{\bar{\text{q}}}_{k}^{nb}\right)}{\partial {\bar{\text{q}}}_{k}^{nb}}=\text{K}\;{\text{R}}^{cb}\Psi \left({\bar{\text{q}}}_{k}^{nb},{\text{Z}}_{j}^{n}-{\text{b}}_{k}^{n}\right)={\text{H}}_{k}^{\text{vis}}$$

The Jacobian matrix related to the translational part of the state vector can be written:

(28) $${\text{H}}_{\text{T}\;k}^{\text{vis}}=\left[\begin{array}{cccccc} {\left(\frac{\partial {\text{y}}_{j,k}^{\text{vis}}}{\partial {\text{b}}_{k}^{n}}\right)}^{\left(1\right)} & 0_{3\times 1} & {\left(\frac{\partial {\text{y}}_{j,k}^{\text{vis}}}{\partial {\text{b}}_{k}^{n}}\right)}^{\left(2\right)} & 0_{3\times 1} & {\left(\frac{\partial {\text{y}}_{j,k}^{\text{vis}}}{\partial {\text{b}}_{k}^{n}}\right)}^{\left(3\right)} & 0_{3\times 1} \end{array}\right]$$

where ${\left(\frac{\partial {\text{y}}_{j,k}^{\text{vis}}}{\partial {\text{b}}_{k}^{n}}\right)}^{\left(m\right)}$ denotes the *m*-column of $\frac{\partial {\text{y}}_{j,k}^{\text{vis}}}{\partial {\text{b}}_{k}^{n}}$:

(29) $$\frac{\partial {\text{y}}_{j,k}^{\text{vis}}}{\partial {\text{b}}_{k}^{n}}=-\text{K}\;{\text{R}}^{cb}{\text{R}}_{k}^{bn}$$

The visual measurement noise covariance matrix ***R****_vis_* can be written as:

(30) Rvis=I2Nf×2Nf⋅σ2vis

where the standard deviation *^vis^σ* measures the uncertainty of the Harris corner finder [33]. We chose the value *^vis^σ* = 0.75 pixel, rather than the more optimistic value suggested in [33] (*^vis^σ* = 0.1 pixel), which gave rise to a more stable filter in our experiments.

### 2.4. Experimental Validation

Eight tracking experiments, each lasting 60 s, were conducted by moving the sensor unit assembly of Figure 1 freely by hand in all six DOFs, with the constraint to keep the chessboard area always within the camera FOV. The angular velocities were up to 40°/s and the linear accelerations were up to 0.6 m/s^2^. An additional tracking experiment was performed by moving the sensor unit assembly 1° at a time.

The IMU sensors were calibrated using the in-field calibration techniques described in [34]. In particular, the gyroscope was compensated for the initial bias value by taking the average of its output during a rest period of 1 s, just before the IMU motion started (bias-capture procedure).

The following filtering methods were tested: the purely IMU-based method of orientation estimation (Section 2.1); the purely vision-based method of pose estimation (Section 2.2); and the two methods of sensor fusion named DLT-based EKF (Section 2.3.1) and error-driven EKF (Section 2.3.2). In all cases no gating technique was implemented in the EKFs to detect outliers due to mismatched features in consecutive image frames. The sensor data acquired during the tracking experiments were analyzed for the off-line validation study in five different filtering scenarios: (a) inertial/magnetic sensor measurements from the IMU were ignored by the filters; (b) inertial/magnetic sensor measurements from the IMU were assimilated in the filters; (c) the magnetic sensor measurements from the IMU were ignored by the filters; (d) gyro bias was not compensated by bias capture, in the situation when magnetic sensor measurements from the IMU were ignored by the filters; (e) a mechanism of intentional damage to the integrity of visual information was implemented and inertial/magnetic sensor measurements were assimilated by the filters. The rationale behind (c) was to stress the importance of magnetic sensor measurements for heading stabilization. The rationale behind (d) was to urge the capability of the proposed sensor fusion methods to accommodate slight imperfections that are typical of inertial sensors. Finally, the rationale behind (e) was to assess the tracking robustness of the sensor fusion methods against visual gaps. The mechanism for degrading the visual information made available to the DLT-based EKF and the error-driven EKF was implemented as follows: for each incoming image frame, a random sample of visual features with size randomly selected from 0 (*i.e*., no deletions occurred) to the maximum number tolerated by each filter (*i.e*., nine for the error-driven EKF and three for the DLT-based EKF) was discarded by setting the corresponding rows of the Jacobian matrix **H***^vis^* to zero (this trick allowed preventing the information associated with the selected features to influence the filtering process); at the next image frame, number and identity of the removed visual features were due to change independently based on the chosen random selection process. The filter parameter setting reported in Table 1 was chosen.

The reference data were interpolated using cubic splines to the time instants when inertial/magnetic and visual measurements were made. Standard conversion formulae were then used to convert the reference and estimated quaternions in the corresponding Euler angles. The performance assessment was based on the root mean square errors (RMSEs) of the estimated roll, pitch and yaw angles. Moreover, the error quaternion $\Delta \bar{\text{q}}={\bar{\text{q}}}_{\text{ref}}^{-1}⊗\hat{\bar{\text{q}}}$ represented the estimated rotation needed to bring the estimated body frame into {**b**}: the scalar component of Δ***q̅***, namely Δ*θ* = 2 cos^−1^(Δ*q*_4_) was used to compute the orientation RMSE. The RMSE of the estimated position was computed separately for each coordinate axis (*e_X_*, *e_Y_* and *e_Z_*) and as a total position error $e_{T}=\sqrt{e_{X}^{2}+e_{Y}^{2}+e_{Z}^{2}}$. Finally, the RMSE values calculated in the eight tracking experiments were summarized using mean value and standard deviation (SD).

The filtering algorithms were implemented using Matlab; the experimental validation was carried out in off-line conditions. Since the 10-dimensional state vector was the same for either the DLT-based EKF or the error-driven EKF, the operations involved in the prediction stage were exactly the same, which took (approximately) 1 ms in the current implementation (standard laptop, 2.2 GHz clock frequency). Another common element was the vector matching process for the sensed acceleration and magnetic field vectors, which required 1 ms, while the computation of the inertial/magnetic Jacobian matrix took approximately 1 ms. The difference between the two EKFs was in the visual measurement equations: in the DLT-based EKF 10 measurement channels were deployed, in contrast with the 24 measurement channels needed by the error-driven EKF. The computation of the visual features required 14 ms in both filters, which included state propagation and prediction. In the DLT-based EKF, the DLT method was implemented at each iteration cycle, followed by the update of the time-varying measurement noise covariance matrix in Equation (24); conversely, in the error-driven EKF the computation of the visual Jacobian matrix—see Equations (27–29)—was needed at each iteration cycle. In conclusion, both filters would require 16 ms for each iteration cycle when an image frame was available for processing. The purely vision-based method was more computationally expensive (approximately, 28 ms), mainly because of the need for the pyramidal implementation of the KLT tracker. The purely IMU-based method took about 2 ms for iteration cycle.

## 3. Experimental Results

The RMSE values of the eight tracking experiments are summarized in mean value ± SD in Table 2, when all tested filtering methods are based on visual measurements only, and in Tables 3–5, where visual measurements are fused with inertial/magnetic measurements: in particular, Tables 4 and 5 report the summary statistics of the performance metrics when magnetic measurements are prevented from influencing the filtering process—the conditions under which data in Table 4 are produced differ from those valid for Table 5 depending whether the gyro bias capture is enabled (Table 4) or not (Table 5). The label TF, *i.e.*, Tracking Failure indicates the inability of the error-driven EKF to successfully complete the tracking task when the inertial/magnetic measurements are not integrated within the filter. The label N/A, *i.e.*, Not Available indicates the inability of the purely-IMU based method of orientation estimation to do positioning.

**Figure 5.**
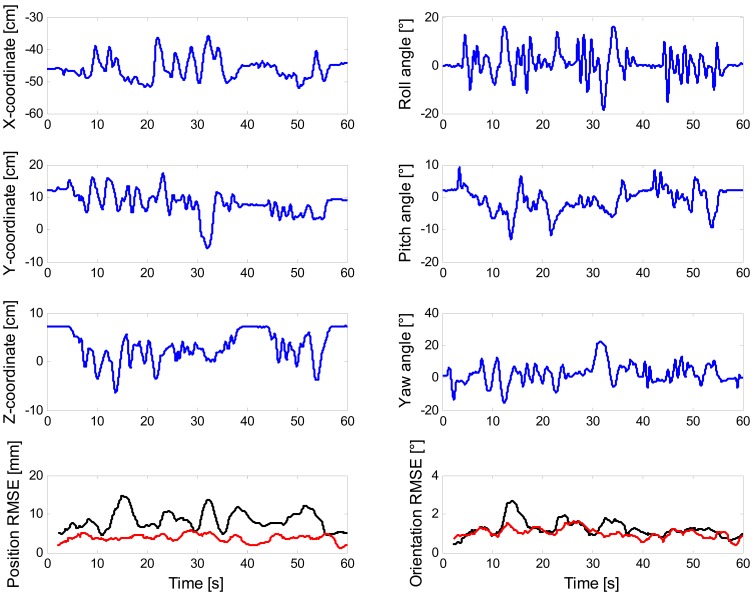
For one of the eight tracking experiments, plots of reference position and Euler angles of the body pose, together with plots of the position and orientation RMSE. For the sake of visualization, the RMSE values are computed over moving average windows of duration 5 s (DLT-based EKF, in red; error-driven EKF, in black).

The representative plots in Figure 5 are produced by running the DLT-based EKF and the error-driven EKF using sensor data from one of the eight tracking experiments in the scenario (b).

**Figure 6.**
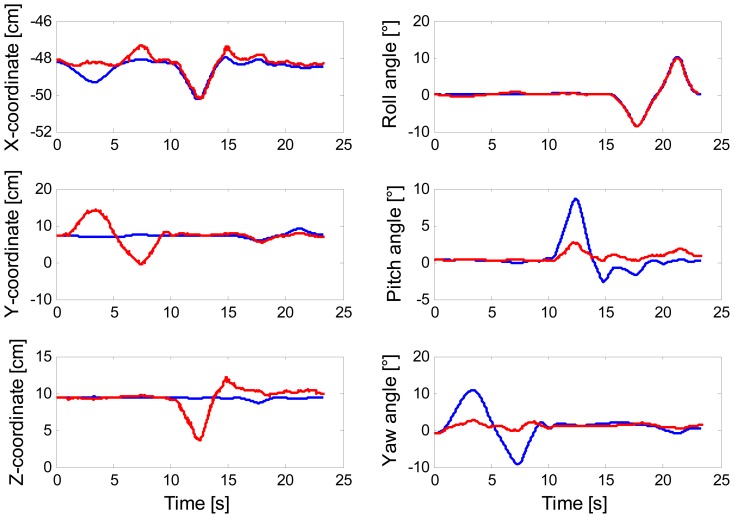
Reference and filtered position and Euler angles that are produced using the error-driven EKF (pure vision). Reference data in blue; filtered data in red.

**Figure 7.**
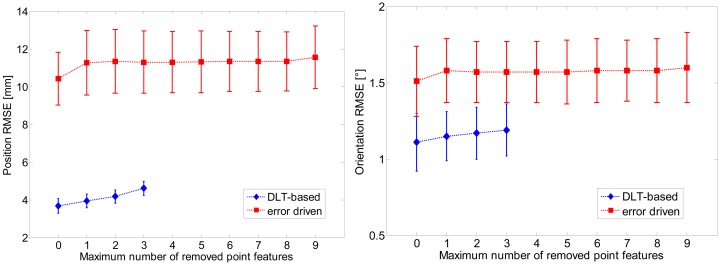
Error plots of the position and orientation RMSEs *versus* the number of image feature points that are removed at each iteration during processing by either the DLT-based EKF or the error-driven EKF (see text).

The plot of Figure 6 concerns the results of tracking one rotational DOF at a time, when the error-driven EKF runs in the scenario (a). Finally, the results of eroding the amount of visual information made available to the filtering methods are presented in Figure 7.

## 4. Discussion

In this paper positioning is not attempted using inertial/magnetic sensors alone, as it is done, e.g., in pedestrian navigation systems, when the IMU is attached to the foot. The exploitation of biomechanical constraints that concern the dynamics of human walking allows indeed mitigating the error growth incurred in the double-time integration process of gravity-compensated acceleration components: for instance, the cubic-time growth of positioning errors can be broken down to a linear-time growth by implementing zero-velocity updates (ZUPT) at the times when the foot is detected steady during walking [35]. This approach cannot be pursued in general, and in particular when the tracked motions are too slow and rest periods for ZUPT are infrequent, if any, which is the case in the tracking experiments discussed in this paper. In other words, positioning is possible in our experimental setup only because of the availability of monocular vision, provided that we can properly deal with the scale ambiguity in the translational ego-motion. The DLT-based EKF using vision alone and the purely vision-based method are characterized by the same accuracy of pose estimation in the experimental trials of this paper, as shown in Table 2; it is worth noting that, when inertial/magnetic measurements are incorporated in the filter, the predictive mechanism implemented in the DLT-based EKF allows it to perform the feature tracking task with the same efficiency as the KLT algorithm and much lower computational costs.

However, the informative contribution of the inertial/magnetic or just the inertial measurements to the DLT-based EKF is not relevant to boost the accuracy of pose estimation—for slow tracked motions, the DLT-based visual measurements are sufficient to obtain very accurate pose estimates—see Tables 2–5.

In contrast to the DLT-based EKF, the error-driven EKF benefits greatly from the integration of inertial/magnetic or from inertial measurements (to a lesser extent), without which it fails in the experimental trials of this paper. The error-driven EKF performs better, or even much better, than the purely IMU-based method in terms of attitude estimation accuracy, while yielding quite accurate estimates of position too. However, some problems of the error-driven EKF are raised, especially when magnetic measurements are not incorporated in the filtering process, which are not shown by the DLT-based EKF. Our explanation is that providing the sensor fusion method with direct measurements of the quaternion and translation vector of interest is much more informative than relying on visual projection errors as the error-driven EKF does.

The value of incorporating the magnetic sensor measurements in the sensor fusion process is assessed by analyzing the data reported in Tables 3–5. Since the visual measurements are highly informative on all six DOFs, the DLT-based EKF performs accurately even in the experimental scenarios (c) and (d) (Tables 4 and 5). Conversely, the error-driven EKF suffers substantially from lacking the magnetic sensor information, although the visual measurements allow somewhat mitigating the error growth in the orientation estimates. Nonetheless, the positioning accuracy is due to degrade significantly, especially in the experimental scenario (d), which is reflected in the quite high SDs attached to the RMSE average values in Tables 4 and 5.

The reason is that the error-driven EKF may suffer from gross mismatches between estimated and reference poses. In practice, wrong state vector estimates are produced, which do not preclude however the system from successfully tracking the image point features. This is a good instance of the problem of ambiguous/multiple solutions to the pose estimation problem. As discussed in [36,37], the motion of a planar target seen from perspective views can result ambiguous even if four or more coplanar points are used to generate the 2D/3D correspondences. A typical ambiguity problem is represented by the rotation/translation coupling [37] in which yaw or pitch angle variations are interpreted as translational displacements along the *Y*- or *Z*-axis, respectively, as shown in Figure 6—see Figure 1 for interpreting the meaning of the axes: changes of the yaw angle are wrongly interpreted as motion occurring along the *Y*-axis, in the same way as changes of the pitch angle are misleadingly interpreted as motion occurring along the *Z*-axis. Moreover the state parameter values that minimize the projection errors may be quite different from the physical orientation and translation from {**b**} to {**n**}. This problem is due to the non-linear nature of the least-square method used by the error-driven EKF to generate the pose from the projection errors, which is prone to local minima. Visuo-inertial integration is a suitable means to deal with the problem of ambiguous/multiple solutions: the error-driven EKF is indeed capable of correctly disambiguating critical motions thanks to the IMU measurements, especially when measurements from the magnetic sensor are integrated and gyro bias is properly compensated by the bias capture procedure, as shown in Table 3.

The visual sabotage implemented in this paper is not as extreme as permanent losses of image point features would be, such as those occurring in case of occlusions, or when the ego-motion is so fast that part or all of the chessboard area escapes the camera FOV. We simply limit to randomly reduce number and location of coplanar feature points, sometimes even below the minimum number theoretically needed for pose estimation. The data reported in Figure 7 demonstrates the superiority, in terms of visual robustness terms, of the error-driven EKF over the DLT-based EKF. In fact, the former filter can tolerate reductions down to zero of the image point features without experiencing tracking losses of any kind while the latter absolutely needs a minimum number of six image point features. In addition, the RMSE values of the DLT-based increase progressively with the number of removed features, in contrast to the RMSE values of the error-driven EKF.

The main problem experienced in regard of loss of vision is as follows: since it is only the vision that does positioning, the position estimates tend to diverge fast when the system is blind, visually speaking. While the orientation estimates are continuously and accurately provided by the inertial/magnetic sensors, it is this diverging trend that explains why projection errors may rapidly grow to an extent that makes impossible for the system to maintain the track on the chosen fiducial markers. To make matters worse, we have decided not to implement any mechanism for monitoring the filter divergence based on the number of visual features registered, or any re-initialization procedure in case of divergence [38]: a Kalman-based filter would be capable, in principle, of recovering tracking losses of short duration using either the information on the motion trajectory captured by the dynamic model or the information from the inertial/magnetic sensors.

## 5. Conclusions

In this paper two approaches to fuse visual and inertial/magnetic measurements have been considered and correspondingly two EKFs have been developed to track the ego-motion in all six DOFs. They were analyzed with the aim to elucidate how the visual and inertial/magnetic measurements cooperate together and to which extent they do for ego-motion estimation. The two filters perform differently in terms of accuracy and robustness: in the DLT-based EKF the visual measurements seem to have a higher informational content as compared to the inertial/magnetic measurements, and the overall system shows remarkably good accuracy in estimating all six DOFs; conversely, in the error-driven EKF the inertial/magnetic measurements are fundamental for the correct operation of the filter, and the overall system can thus gain in robustness against loss of visual information, at the expense of accuracy in estimating all six DOFs. Moreover, the strategy of sensor fusion is interesting in other respects: on the one hand, the DLT-based EKF takes advantage of the inertial/magnetic measurements since visual features can be tracked without using tools like the KLT, which are computationally time-consuming; on the other hand, the error-driven EKF does positioning only because of its capability of exploiting the projection errors of the image point features.

That magnetic sensor measurements can be helpful to stabilize heading is highlighted in our results, although this statement cannot be overemphasized given the difficulties of motion tracking in magnetically perturbed environments [39]. Another limitation of the present work is that we have not considered the effects of fast motions on the filter behavior. Actually, we have implemented vector selection schemes for accelerometer and magnetic sensor measurements, as done, e.g., in [24]; however, due to the benign nature of the tracked motions and the magnetic environment surrounding the IMU, they were substantially inactive during all tracking experiments described in this paper. A possibility to deal with magnetically perturbed environments would be to augment the state vector with the magnetic disturbance as done, e.g., in [39]; a possibility to deal with aggressive movements would be to modify the state vector by including angular velocity and linear acceleration into it [2,18,40]. Both possibilities are technically feasible in our approach, and they are left for our ongoing work. We plan to improve this work in several other respects: in particular we intend to remove the limitations of working with fixed calibration patterns like the chessboard by exploiting natural features that are usually present in unprepared environments, paving the way to the implementation of an SFM system. Although this effort may greatly complicate the feature extraction/tracking steps, faster and more natural ego-motions would be considered in our experimental scenarios.

In conclusion, in this paper we proposed two different models of visual measurements to be used within Kalman-based filters that also incorporate inertial/magnetic measurements for estimating the ego-motion of a hand-held IMU/camera sensor unit. The two proposed EKFs were off-line analyzed in different experimental conditions: the DLT-based EKF was more accurate than the error-driven EKF, less robust against loss of visual features, and equivalent in terms of computational complexity. Orientation RMSEs of 1° (1.5°) and position RMSEs of 3.5 mm (10 mm) were achieved in our experiments by the DLT-based EKF (error-driven EKF). By contrast, the purely IMU-based EKF achieved orientation RMSEs of 1.6°.

## Figures and Tables

**Table 1.** Parameter tuning.

**Process noise**	
*σ_g_* [°]	0.40
*^α^σ* [m/s^2^]	0.05
**Measurement noise**	
*σ_h_* [mGauss]	2
*σ_α_* [m*g*]	10
*σ_θ_* [° ]	0.05
*σ_b_*[mm]	1
*^vis^σ* [pixel]	0.75
**Vector selection**	
*λ_h_* [mGauss]	20
*λ*_dip_ [°]	5
*λ_g_* [m*g*]	20

**Table 2.** Summary statistics of the performance metrics in the scenario (a).

	**Purely-vision based**	**DLT-based EKF**	**Error-driven EKF**
Yaw, °	0.45 ± 0.08	0.40 ± 0.04	TF
Pitch, °	0.64 ± 0.10	0.66 ± 0.13	TF
Roll, °	0.78 ± 0.14	0.73 ± 0.09	TF
Orientation, °	1.11 ± 0.16	1.09 ± 0.19	TF
*X*, mm	1.55 ± 0.42	1.54 ± 0.31	TF
*Y*, mm	2.67 ± 0.59	2.88 ± 0.43	TF
*Z*, mm	4.45 ± 0.71	2.14 ± 0.32	TF
Position, mm	5.45 ± 0.76	3.95 ± 0.52	TF

**Table 3.** Summary statistics of the performance metrics in the scenario (b).

	**Purely-IMU based**	**DLT-based EKF**	**Error-driven EKF**
Yaw, °	1.04 ± 0.27	0.41 ± 0.06	0.81 ± 0.16
Pitch, °	0.76 ± 0.19	0.63 ± 0.10	0.78 ± 0.31
Roll, °	0.96 ± 0.15	0.78 ± 0.10	0.92 ± 0.13
Orientation, °	1.61 ± 0.29	1.08 ± 0.13	1.46 ± 0.25
*X*, mm	N/A	1.37 ± 0.41	2.59 ± 0.56
*Y*, mm	N/A	2.82 ± 0.48	6.72 ± 1.20
*Z*, mm	N/A	1.96 ± 0.24	6.64 ± 2.41
Position, mm	N/A	3.40 ± 1.10	10.00 ± 1.75

**Table 4.** Summary statistics of the performance metrics in the scenario (c).

	**Purely-IMU based**	**DLT-based EKF**	**Error-driven EKF**
Yaw, °	2.16 ± 2.03	0.43 ± 0.06	2.34 ± 1.81
Pitch, °	0.80 ± 0.16	0.65 ± 0.09	0.84 ± 0.20
Roll, °	1.29 ± 1.40	0.81 ± 0.11	0.81 ± 0.15
Orientation, °	3.00 ± 1.95	1.10 ± 0.15	2.72 ± 1.63
*X*, mm	N/A	1.48 ± 0.44	4.47 ± 2.01
*Y*, mm	N/A	3.03 ± 0.32	20.67 ± 13.4
*Z*, mm	N/A	2.01 ± 0.29	5.89 ± 2.13
Position, mm	N/A	3.90 ± 0.51	22.22 ± 13.0

**Table 5.** Summary statistics of the performance metrics in the scenario (d).

	**Purely-IMU based**	**DLT-based EKF**	**Error-driven EKF**
Yaw, °	29.93 ± 0.90	0.41 ± 0.06	3.07 ± 0.62
Pitch, °	1.01 ± 0.27	0.69 ± 0.09	1.20 ± 0.51
Roll, °	1.16 ± 0.17	0.77 ± 0.10	1.00 ± 0.16
Orientation, °	29.99 ± 0.91	1.08 ± 0.13	3.41 ± 0.65
*X*, mm	N/A	1.36 ± 0.41	4.92 ± 1.58
*Y*, mm	N/A	2.82 ± 0.48	24.27 ± 6.33
*Z*, mm	N/A	1.97 ± 0.24	9.19 ± 4.80
Position, mm	N/A	3.71 ± 0.60	26.79 ± 6.27
